# Impact of the Medicare hospital readmissions reduction program on vulnerable populations

**DOI:** 10.1186/s12913-019-4645-5

**Published:** 2019-11-14

**Authors:** Yunwei Gai, Dessislava Pachamanova

**Affiliations:** 1 Associate Professor, Economics Division, Babson College, 231 Forest Street, Babson Park, MA 02457 USA; 2 Professor, Mathematics and Sciences Division, Babson College, 231 Forest Street, Babson Park, MA 02457 USA

**Keywords:** Hospital readmissions reduction program (HRRP), 30-day hospital readmissions, Vulnerable population, Difference-in-differences (DD), Triple difference (DDD), Health policy

## Abstract

**Background:**

The Hospital Readmissions Reduction Program (HRRP) was established by the 2010 Patient Protection and Affordable Care Act (ACA) in an effort to reduce excess hospital readmissions, lower health care costs, and improve patient safety and outcomes. Although studies have examined the policy’s overall impacts and differences by hospital types, research is limited on its effects for different types of vulnerable populations. The aim of this study was to analyze the impact of the HRRP on readmissions for three targeted conditions (acute myocardial infarction, heart failure, and pneumonia) among four types of vulnerable populations, including low-income patients, patients served by hospitals that serve a high percentage of low-income or Medicaid patients, and high-risk patients at the highest quartile of the Elixhauser comorbidity index score.

**Methods:**

Data on patient and hospital information came from the Nationwide Readmission Database (NRD), which contained all discharges from community hospitals in 27 states during 2010–2014. Using difference-in-difference (DD) models, linear probability regressions were conducted for the entire sample and sub-samples of patients and hospitals in order to isolate the effect of the HRRP on vulnerable populations. Multiple combinations of treatment and control groups and triple difference (DDD) methods were used for testing the robustness of the results. All models controlled for the patient and hospital characteristics.

**Results:**

There have been statistically significant reductions in readmission rates overall as well as for vulnerable populations, especially for acute myocardial infarction patients in hospitals serving the largest percentage of low-income patients and high-risk patients. There is also evidence of spillover effects for non-targeted conditions among Medicare patients compared to privately insured patients.

**Conclusions:**

The HRRP appears to have created the right incentives for reducing readmissions not only overall but also for vulnerable populations, accruing societal benefits in addition to previously found reductions in costs. As the reduction in the rate of readmissions is not consistent across patient and hospital groups, there could be benefits to adjusting the policy according to the socioeconomic status of a hospital’s patients and neighborhood.

## Background

The Hospital Readmissions Reduction Program (HRRP) was established by the 2010 Patient Protection and Affordable Care Act in an effort to reduce excess hospital readmissions, lower health care costs, and improve patient safety and outcomes. It is part of the Centers for Medicare and Medicaid Services (CMS) value-based programs designed for better care for individuals, better health for populations and lower costs (e.g., Hospital Value-Based Purchasing Program, Hospital Acquired Conditions Reduction Program, and Value Modifier (VM) Program). Launched in October 2012, the HRRP penalizes hospitals that have higher than expected readmission rates by reducing Medicare payments. The HRRP targets specific conditions. The initial target conditions were acute myocardial infarction (AMI), heart failure (HR), and pneumonia (PN). In 2015, several target conditions were added, including chronic obstructive pulmonary disease (COPD), coronary artery bypass graft (CABG) surgery, and elective primary total hip arthroplasty and/or total knee arthroplasty (THA/TKA) [1].

From early on, there have been concerns about whether the HRRP can lead to unintended consequences for patients of low socioeconomic status and hospitals serving them. One study [2], analyzing pre-HRRP data from July 1, 2008, to June 30, 2011, noted that hospitals with the highest percentage of Medicaid or uninsured patients are ‘…30 percent more likely to have 30-day hospital readmission rates above the national average…, and will therefore be disproportionately impacted by the HRRP.’ Another study [3] analyzed 2009 Medicare administrative claims data and assessed the potential impact of the HRRP on hospitals with a high percentage of patients on both Medicare and Medicaid. Noting that dual-eligible patients experienced higher readmission rates for all three target conditions (AMI, HR, and PN), the authors argued that HRRP penalties would fall disproportionately on such hospitals, which would affect vulnerable populations.

Researchers, using 2009–2012 Medicare data, found that ‘[h]ospitals with high readmission rates may be penalized to a large extent based on the patients they serve [4].’ Their concerns were echoed by others [5], who found, using 2007–2010 data, that a substantial amount (58%) of variation in national hospital readmission rates can be explained by community factors as measured by county characteristics. A recent study [6] used Medicare administrative claims data from July 2007 to June 2010 to analyze the range and distribution of readmissions by patients’ socioeconomic status. They found little differences in readmission rates between hospitals serving large and small proportions of patients with low socioeconomic status.

These conclusions, drawn from data before the HRRP, are informative; but it is unclear whether the rates of readmission reduction after the HRRP took effect are similar across these hospitals. As the authors pointed out in the conclusion of [6], ‘…much remains to be learned about … whether such hospitals are able to improve at the same rate as others.’

Empirical studies with data collected after the HRRP launched found mixed results when it comes to low socioeconomic status patients and the hospitals serving them. A 2017 study using 2008–2014 Virginia hospital data found a decrease in AMI readmissions as well as no changes in the age, race/ethnicity, health status, and socioeconomic status of patients admitted for AMI [7]. Bazzoli, Thompson and Waters, using 2013–2014 national-level data for 2720 hospitals, found evidence that safety-net hospitals incurred higher HRRP penalties than non-safety-net hospitals but that the increase in their penalty rate did not significantly affect their total margins, as safety-net hospitals appeared to rely on non-patient care revenues to offset the higher penalties [8]. These additional funds, however, may come from sources that address important community needs, such as local health promotions or other community benefit programs.

On the whole, the HRRP has been deemed a success at both the local and the national levels. Studies have found an overall decrease in readmission rates [9–14], positive spillover effects when it comes to non-targeted conditions and patients not on Medicare [7, 15, 16], as well as ‘no evidence that hospitals delay readmissions, treat patients with greater intensity, or alter discharge status in response to the HRRP’ [7] and no significant differences between not-for-profit and proprietary hospitals [17]. The program has saved the CMS millions of dollars through reimbursement cuts as penalties as well as lower reimbursement to hospital visits because of prevented readmissions [18].

The goal of this study is to examine whether observations about the effect of the HRRP on readmission rates translate to different types of populations. We especially focus on vulnerable populations with the following definitions:

1. Patients served by hospitals that serve a high percentage of low-income patients.
2. Patients served by hospitals that serve a high percentage of Medicaid patients.
3. Patients of low socioeconomic status measured by the median household income in the Zip Code of their residence.
4. High-risk patients whose admission is at the highest quartile of the Elixhauser comorbidity index score.

Definitions 1 and 2 describe a hospital’s socioeconomic status, whereas definitions 3 and 4 describe a patient’s socioeconomic status and health condition in terms of comorbidity and mortality risk. Analyzing outcomes based on these different perspectives may potentially have different implications. For example, it is possible for hospitals located in high-income areas to serve a large percentage of low-income patients. Based on a large national dataset and explicitly considering differences in outcomes for different hospitals or patients in terms of multiple measures of socioeconomic and health status, our study can shed light on the impact of HRRP across the spectrum of hospital and patient populations.

## Methods

This study used the difference-in-difference (DD) method, a quasi-experimental design to compare the pre-HRRP (i.e., 2010–2011) differences in readmission rates between treatment and control groups with their post-HRRP (i.e., 2012–2014) differences. Two control/comparison groups were identified to test the robustness of our results. Data in this study was obtained from the Healthcare Cost and Utilization Project (HCUP), with all identifying information removed to protect the privacy of individual patients, physicians, and hospitals. An exemption is granted by the Babson College Institutional Review Board.

### Data source

The study uses the Nationwide Readmissions Database (NRD) for 2010–2014 covering the period before and after the launch of the HRRP. This is a large dataset that includes all discharges from community hospitals in 27 states, excluding rehabilitation or long-term acute care hospitals. The annual NRD data has 14 to 17 million discharges. The large sample allows us to conduct an in-depth analysis of the impact of HRRP on the patient and hospital subgroups. As mentioned, some studies [6] are concerned with the effect of the HRRP on some types of vulnerable populations but assess the impact using data from before the launch of the HRRP. Other studies [7, 14] are more recent but focus on smaller state-level data sets. Our choice of study period and national level data overcomes these two limitations. Similarly to studies [7, 14], we defined the two years before the launch of the HRRP (2010–2011) as the ‘pre-HRRP’ period and the three years after the launch of the HRRP (2012–2014) as the ‘post-HRRP’ period, during which time HRRP targeted three conditions among Medicare patients (AMI, HF and PN). A limitation of NRD is that its hospital identifiers cannot be linked across years or linked to other databases. A new set of unique IDs is created each year in NRD.

### Outcome and exposure variables

We followed CMS reports [19, 20] to construct our analytical samples and 30-day readmission measures. For each of the index hospitalizations (AMI, HF or PN) based on a patient’s principle ICD-9 codes as outlined in the CMS reports, a corresponding 30-day readmission indicator was set to one if readmission (for any conditions, except planned visits or procedures) occurred within 30 days of discharge. Planned readmissions, defined as ‘intentional readmission within 30 days of discharge from an acute care hospital that is a scheduled part of the patient’s plan of care’ do not count as readmissions [20]. Following the CMS definition, we excluded admissions for patients who died during hospitalization, were discharged against medical advice, or were transferred to another acute care facility. In addition, we created indicators for any-target readmissions and non-target readmissions. Any-target readmissions took the value of one if a patient had an either AMI, HF or PN 30-day readmission; and zero otherwise. We use this measure to evaluate the HRRP’s impact on the three targeted conditions as a whole. If a readmission was within 30 days of discharge not from any targeted condition, then the non-target readmission indicator was set to one; and zero otherwise. We use this measure to evaluate HRRP’s impact on non-targeted readmissions.

The primary exposure was whether a patient was directly affected by the HRRP policy (i.e., treatment group) or not directly affected (i.e., control group) based on their insurance status, age, and medical conditions. The readmission measures were calculated for the following five treatment groups among Medicare patients aged 65 and up: each of the three targeted conditions (AMI, HF or PN) separately, the three targeted conditions as a whole, as well as non-target conditions. For each targeted or non-targeted conditions, we created two control groups which the HRRP should not affect directly: (1) readmissions among Medicare patients aged 65 and up with the gastrointestinal (GI) condition [7, 10, 14] as identified by MS-DRG codes of 329–331, 377–379, and 391–392; (2) privately insured patients aged 45 and older with the same condition as the Medicare patients in the corresponding treatment group [7]. In total, there are ten comparisons between treatment and control groups (e.g., Medicare patients aged 65 and up with AMI vs. Medicare patients aged 65 and up with GI; or Medicare patients aged 65 and up with AMI vs. privately insured patients aged 45 and older with AMI).

### Definitions of vulnerable populations

Definitions of population vulnerability in the literature generally depend on the quartile of patient income or insurance types such as Medicaid [2, 3, 8]. We used similar definitions to stratify the data into the four types of vulnerable populations as listed in the Background section.

For the first type of vulnerable population, patients were divided into four groups according to their income level. The NRD data measures a patient’s income level by quartiles of the median household income in their zip code of residence. For example, in 2013, a patient would fall into one of the following quartiles: (Q1) $1 - $37,999; (Q2) $38,000 - $47,999; (Q3) $48,000 - $63,999; and (Q4) $64,000 or more. We will refer to patients in Q1, Q2, Q3, and Q4 as ‘low-income,’ ‘middle-income,’ ‘upper-middle income,’ and ‘high-income’ patients, respectively.

For the second type, hospitals were separated into quartiles based on the percentage of low-income patients relative to total patient volume in each hospital for each year. Q1 contains hospitals whose percentage of low-income patients is in the lowest quartile. Henceforth, we will refer to them as ‘high-income’ hospitals. Q4 contains hospitals whose percentage of low-income patients is in the top quartile. We will refer to them as ‘low-income’ hospitals. Groups Q2 and Q3 correspond to the second and the third quartiles, and will be referred to as ‘middle-income’ and ‘upper-middle income’ hospitals, respectively. The quartiles were recalculated each year because of NRD’s different hospital identifiers over the years.

For the third type, hospitals were separated into quartiles based on their proportion of Medicaid patients. Hospitals in Q1 had the lowest percentage, whereas hospitals in Q4 had the highest percentage. We will refer to the former as ‘low-Medicaid’ hospitals and to the latter as ‘high-Medicaid’ hospitals, respectively.

For the fourth definition of vulnerability, patients in each year were divided into four groups by the quartiles of their Elixhauser mortality index scores [21–23]. The patient severity quartiles were based on the entire patient population in a year, not within a hospital. For example, in 2010, we used the ICD-9 codes to derive the Elixhauser comorbidity index score for each of the 13,907,610 patients. We then divided the patients into four groups by the quartiles of their index scores. Patients in the first quartile had the smallest scores, and will be referred to as ‘low-risk.’ Patients in the top quartile had the largest scores and the most severe comorbidities, and will be referred to as ‘high-risk.’ We repeated this calculation for the other years. This categorization helps to examine the impact of the HRRP by patient severity at the national level.

### Model specification

Different methods have been used to analyze the effect of the HRRP. Several studies rely on difference-in-difference (DD) design [7, 10, 11] or triple difference (DDD) design [7, 14, 16]. Others use descriptive statistics [13] or interrupted time series (ITS) models to compare trends [12]. This study used the DD framework, a popular method in evaluating public policies. We estimated the DD model using the entire sample to measure the program’s overall effect, and sub-samples of patients and hospitals to measure its disparities across vulnerable populations.

Table 1 uses the pre- and post-HRRP readmission rates for a treatment group (Medicare AMI patients) and a control group (Medicare GI patients) to illustrate the DD approach. Medicare AMI readmissions saw a reduction of 1.8 percentage points from 17.4% before the HRRP to 15.6% after the HRRP. During the same period, Medicare GI readmissions decreased by 0.5 percentage points. The difference between readmission rates in the treatment and the control group is thus − 0.013. Alternatively, the difference between Medicare AMI and GI readmission rates before the HRRP is 0.041, whereas the post-HRRP difference is 0.027. Hence the DD estimate is − 0.013 (i.e., 0.027–0.041). If one assumes that the HRRP is responsible for this difference, then it provides an estimate of its impact.

**Table 1:** DD approach illustration with Medicare AMI patients as the treatment group and Medicare GI patients as the control group

Condition/Year	Before HRRP	After HRRP	Difference Over Time
Medicare AMI	0.174	0.156	−0.018***
	(0.0009)	(0.0007)	(0.001)
	*N* = 184,242	*N* = 302,294	*N* = 486,536
Medicare GI	0.133	0.128	−0.005***
	(0.0005)	(0.0004)	(0.0006)
	*N* = 520,669	*N* = 799,687	*N* = 1,320,356
Difference over conditions	0.041***	0.027***	
	(0.001)	(0.001)	
	*N* = 704,911	*N* = 1,101,981	
Difference-in-Difference	−0.013***		
	(0.001)		
	*N* = 1,806,892		

Notes: Unclustered standard errors are reported in parentheses

*** significant at 0.01 level

To control for patient and hospital attributes that could affect readmissions, we estimate the following linear probability models to determine the DD estimates:

1 $$ {Readmission}_{iht}={\beta}_0+{\beta}_1\ {TREAT}_i+{\beta}_2\ {POST}_i+{\beta}_3\ {TREAT}_i\times {POST}_i+\boldsymbol{\theta} \boldsymbol{X}+{\varepsilon}_{iht} $$

where *Readmission*_*iht*_ is an indicator for readmission status for patient *i* at hospital *h* in year *t*. *TREAT*_*i*_ is a dummy variable that equals one if the index hospitalization is targeted by the HRRP; zero if it is in the control group. *POST*_*i*_ is one if patient *i* is from the post-HRRP period, i.e., 2012 and after; zero otherwise. The coefficient of the interaction term between *TREAT*_*i*_ and *POST*_*i*_, *β*_*3*_, is the DD estimate of the impact of the HRRP. *θ* is a vector of coefficients for the patient and hospital attributes listed in the next section. The models controlled for hospital attributes (e.g., teaching status, control type, bed size, and urban-rural location), but not hospital-level fixed effect because NRD’s hospital IDs cannot be linked across years. We also note that hospital attributes could change as a response to HRRP or other policies such as Medicaid expansion.

This DD model is estimated for each of the five types of conditions (AMI, HF, PN, Targeted, Non-Targeted) in order to compare the treatment group with one of the two control groups (Medicare GI or Private Insurance), for a total of ten DD models, each of which is estimated for the entire sample and the 16 quartile-based groups defined previously. Multivariable linear probability models are estimated because they allow coefficient estimates to be interpreted as probability changes and are reliable for measuring average effects [7, 10, 14].

### Covariates

Each of the DD models included patient demographics, socioeconomics, comorbidities, and hospital attributes. Patient demographics included age (measured as dummy variables for age 45–64, 65–74, 75–84, 85 and up), and an indicator for female. Patient socioeconomic status was measured by four quartiles of the median household income for patients’ zip code of residence. We created the Elixhauser mortality index score for each patient as their comorbidity measures [21–23]. We controlled patient location by a six-category urban-rural classification scheme for U.S. counties developed by the National Center for Health Statistics (NCHS). Hospital characteristics included their teaching status, control type (public or private), bed size (small, medium or large), and urban-rural location. In addition, we considered discharge year and quarter for time-fixed effects.

The final data set in our analysis has over 34 million hospitalizations. The number of hospitals in the data ranges from 1715 to 2048 depending on the year. Stata 15 MP (StataCorp LP, College Station, TX) was used for our analysis. All standard errors were robust and clustered at the hospital level for each year.

## Results

**Fig. 1:**
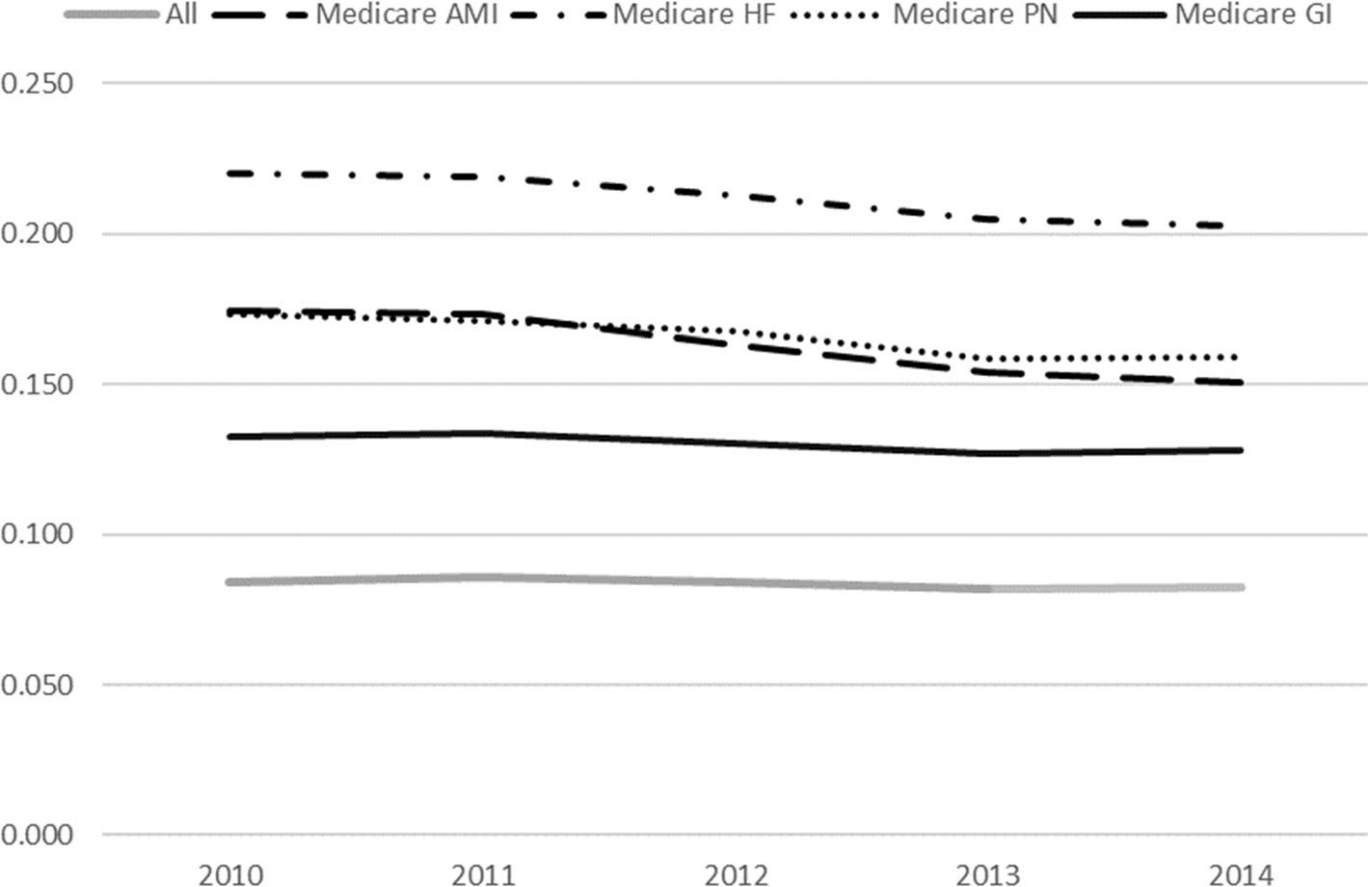
Readmission rates over 2010–2014 for All, Medicare AMI, Medicare HF, Medicare PN and Medicare GI patients

In Fig. 1, we show the trend in readmission rates for 2010–2014 for all patients and conditions in the sample (as a benchmark) as well as separately for Medicare AMI, HF, PN, and GI patients. There is an overall downward trend in 30-day readmission rates over the five-year period. Medicare GI patients, the control group in the DD model, have the lowest readmission rates among the four conditions; however, readmission rates for Medicare GI patients appear to be holding steady or going slightly up in 2014, whereas readmission rates for AMI, HF, and PN have been decreasing. Before 2012 (during the pre-HRRP period), Medicare GI, AMI, HF and PN rates had been decreasing slightly at about the same rate. This observation supports the parallel trend assumption in the DD design which assumes the same trend between the control group and treatment group prior to the policy.

**Fig. 2:**
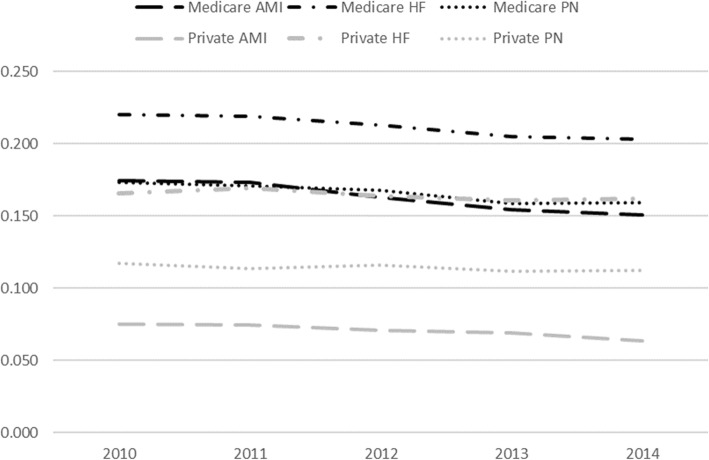
Readmission rates over 2010–2014 for Medicare and private insurance patients with three conditions targeted by the HRRP: AMI, HF, and PN

Figure 2 shows the readmission trends for Medicare patients with the index conditions compared to private insurance patients of 45+ years of age with the same index conditions. The readmission rates are lower for private insurance patients than Medicare patients with the same condition. However, while readmission rates for private insurance patients have held relatively steady, readmission rates for Medicare patients have been decreasing, with the decline starting around 2012. During the pre-HRRP period, Medicare and privately insured patients had a similar rate of change, thus supporting the parallel trend assumption in DD models.

**Fig. 3:**
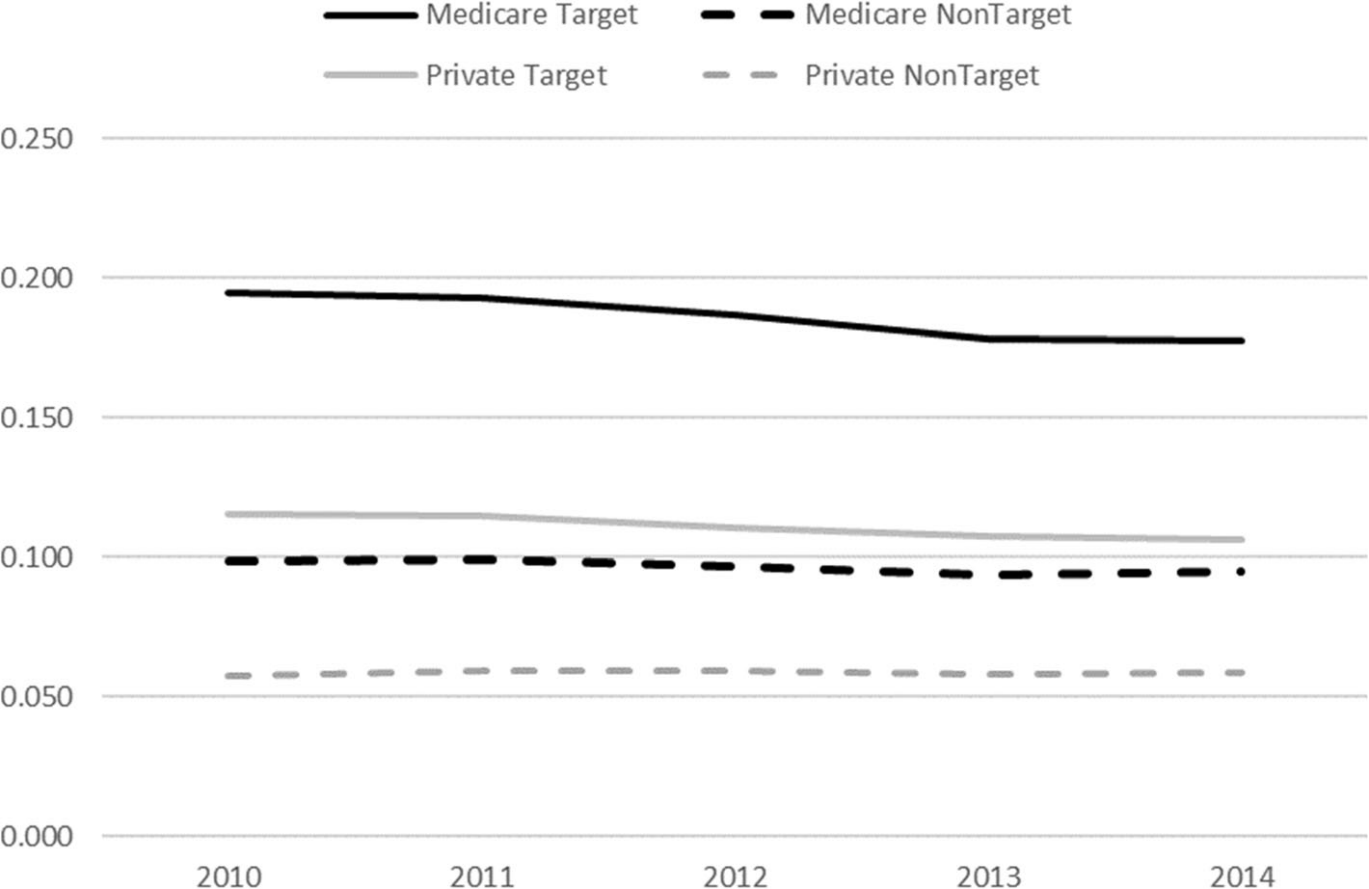
Readmission rates over 2010–2014 for Medicare and private insurance patients for all conditions targeted by the HRRP (Target) and all conditions not targeted by the HRRP (Non-Target)

Figure 3 illustrates the trends for readmission rates for Medicare and privately insured patients for all conditions targeted by the HRRP (‘Target’) and all conditions not targeted by the HRRP (‘Non-Target’). In 2012 and after, all four types of readmission rates decreased, albeit at different rates. The rate of reduction for both Medicare Target and Medicare NonTarget conditions is higher than for private insurance. It is, therefore, possible that there are some spillover effects from the HRRP affecting conditions not targeted by the HRRP as well as private insurance patients.

Tables 2 and 3 summarize the readmission rates for various treatment/control groups and populations in the pre- (i.e., 2010–2011) and post- (i.e., 2012–2014) period. Among Medicare patients, we consistently observe a decline in index readmissions after the HRRP. For example, the Medicare patients’ readmission rate for AMI declined from 17.4 to 15.6%; HF declined from 21.9 to 20.7%; PN declined from 17.2 to 16.2%. The readmission rate for the three targeted conditions went down from 19.4 to 18.1%. During the same time, Medicare patients with GI conditions or non-targeted conditions only had a small decrease from 13.3 to 12.8% and from 9.9 to 9.5%, respectively. The decrease among privately insured patients was even smaller for both targeted and non-targeted readmissions. These observations support the hypothesis that the HRRP has reduced readmissions in index hospitalizations for Medicare patients.

**Table 2:**
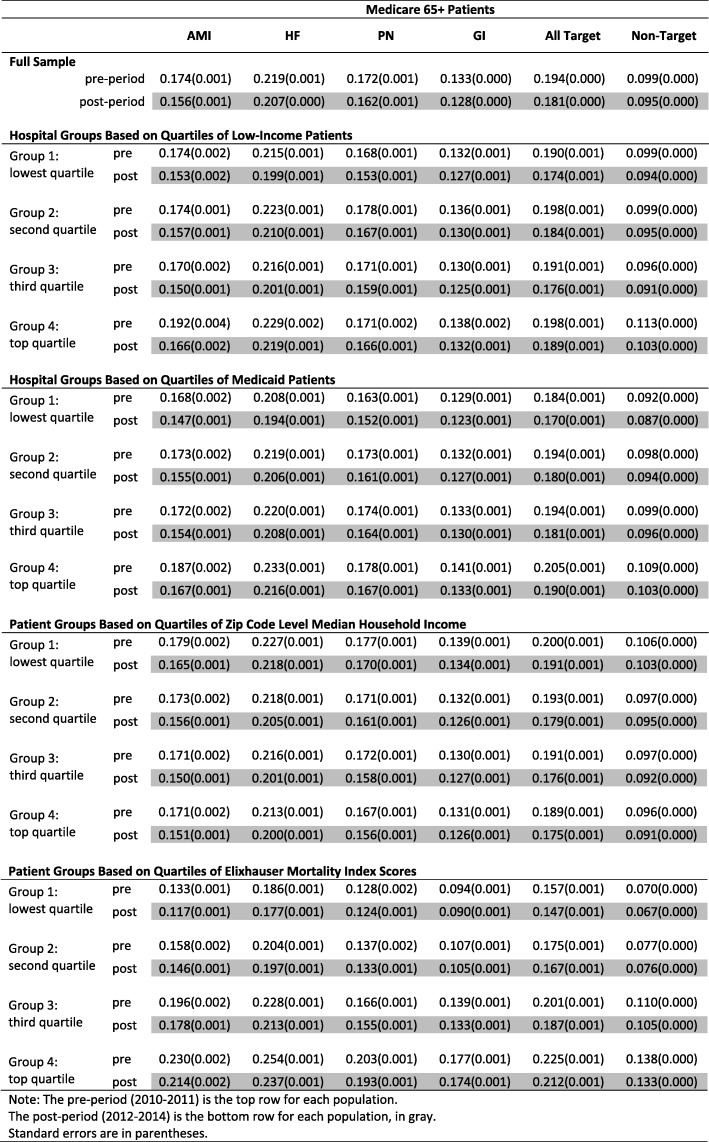
Readmission rates for different Medicare patient groups during the pre- and post- period

Note: The pre-period (2010–2011) is the top row for each population

The post-period (2012–2014) is the bottom row for each population, in gray

Standard errors are in parentheses

**Table 3:**
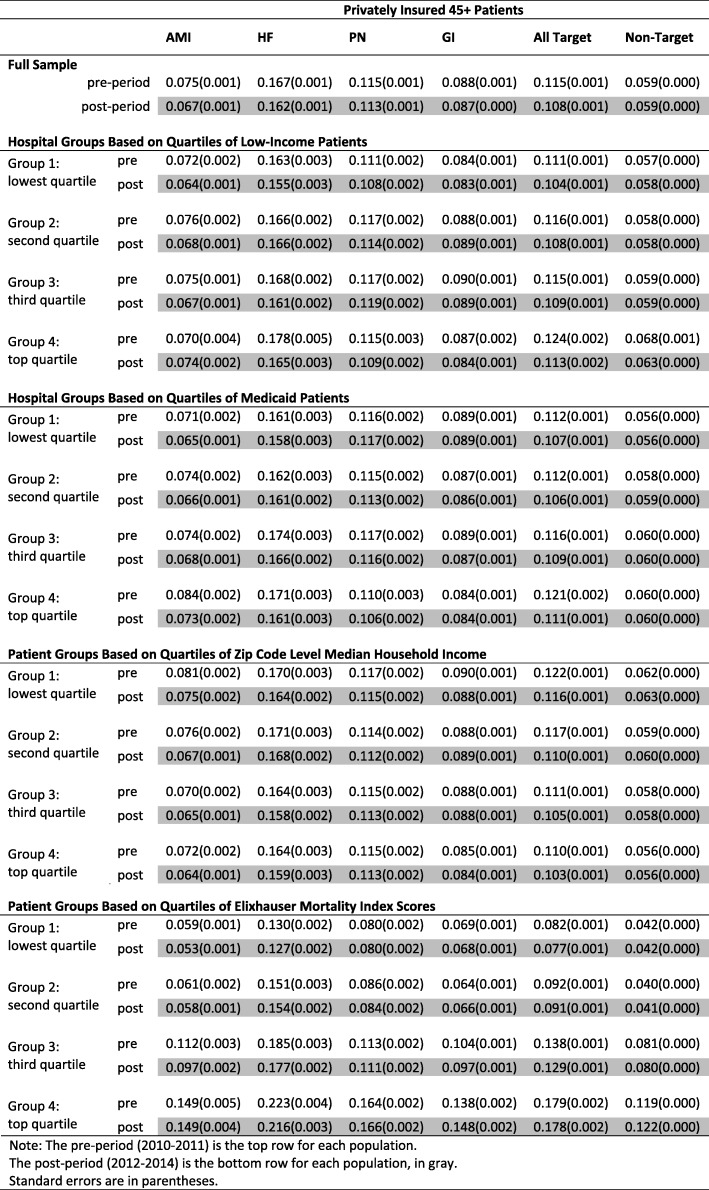
Readmission rates for different private insurance patient groups of age 45+ during the pre- and post-period

Note: The pre-period (2010–2011) is the top row for each population

The post-period (2012–2014) is the bottom row for each population, in gray

Standard errors are in parentheses

The readmission rate for index hospitalizations among Medicare patients decreased across different hospital and patient groups. For example, in hospitals with the smallest proportion of low-income patients (Group 1: lowest quartile in Table 2), the Medicare AMI readmission decreased by 2.1 percentage points, from 17.4 to 15.3%. Hospitals with the largest proportion of low-income patients (Group 4: top quartile in Table 2) had an even larger decrease of 2.6 percentage points, from 19.2 to 16.6%. Readmissions in hospitals of the middle quartiles (Groups 2 and 3 in Table 2) decreased too, by 1.7 and 2.0 percentage points, respectively. Similarly, we observed different rates of decline among other hospital and patient groups and among HF and PN readmissions, but the differences within each group were not large.

It is difficult to identify a consistent pattern when it comes to which hospital or patient group experienced the greatest decline. For example, in hospital groups based on quartiles of low-income patients, Medicare AMI readmissions decreased the most in the top quartile, whereas HF and PN decreased the most in the lowest quartile.

Because vulnerable hospitals and patients had higher readmission rates before the HRRP, they did not catch up with the less vulnerable groups through the end of the period under study, even though in some cases they had larger readmission rate reductions. A case in point is the change in Medicare AMI readmissions. Although low-income hospitals had the largest decrease of 2.6 percentage points within the four income-based hospital groups, their post-HRRP readmission rate (16.6%) was still higher than the readmission rate for high-income hospitals (15.3%). The disparity in readmission rates between vulnerable and less vulnerable groups remained for other index hospitalizations as well.

Trend analysis and descriptive statistics do not control for the heteroscedasticity in patient and hospital attributes, which could affect conclusions. We use DD models to control for these factors. The results for a variety of populations are presented in Table 4.

**Table 4:** DD estimates for various combinations of treatment and control groups and vulnerable populations

Sample	Treatment: Medicare 65+; Control: Medicare GI 65+					Treatment: Medicare 65+; Control: Private Insurance 45+				
	AMI	HF	PN	Target	NonTarget	AMI	HF	PN	Target	NonTarget
Full Sample	-0.012^c^ (0.001)	-0.008^c^ (0.001)	-0.005^c^ (0.001)	-0.008^c^ (0.001)	0.000 (0.002)	-0.009^c^ (0.002)	-0.008^c^ (0.002)	-0.008^c^ (0.002)	-0.007^c^ (0.001)	-0.004^c^ (0.000)
Hospital Groups Based on Quartiles of Low-Income Patients										
Group 1: lowest quartile	-0.014^c^ (0.003)	-0.010^c^ (0.002)	-0.009^c^ (0.002)	-0.011^c^ (0.002)	0.002 (0.005)	-0.010^c^ (0.004)	-0.006 (0.004)	-0.010^c^ (0.004)	-0.008^c^ (0.002)	-0.005^c^ (0.001)
Group 2: second quartile	-0.013^c^ (0.003)	-0.010^c^ (0.002)	-0.005^b^ (0.002)	-0.009^c^ (0.002)	-0.003 (0.004)	-0.010^c^ (0.003)	-0.008^b^ (0.004)	-0.012^c^ (0.003)	-0.009^c^ (0.002)	-0.004^c^ (0.001)
Group 3: third quartile	-0.010^c^ (0.003)	-0.007^c^ (0.002)	-0.004^a^ (0.002)	-0.007^c^ (0.002)	0.003 (0.004)	-0.008^c^ (0.003)	-0.013^c^ (0.004)	-0.006^a^ (0.003)	-0.007^c^ (0.002)	-0.004^c^ (0.001)
Group 4: top quartile	-0.018^c^ (0.005)	-0.006^a^ (0.003)	-0.000 (0.003)	-0.003 (0.003)	0.003 (0.008)	-0.028^c^ (0.006)	-0.001 (0.007)	0.001 (0.005)	-0.002 (0.004)	-0.006^c^ (0.001)
Hospital Groups Based on Quartiles of Medicaid Patients										
Group 1: lowest quartile	-0.013^c^ (0.003)	-0.008^c^ (0.002)	-0.004^a^ (0.002)	-0.008^c^ (0.002)	0.000 (0.005)	-0.012^c^ (0.004)	-0.010^b^ (0.004)	-0.010^b^ (0.004)	-0.009^c^ (0.002)	-0.004^c^ (0.001)
Group 2: second quartile	-0.011^c^ (0.003)	-0.008^c^ (0.002)	-0.007^c^ (0.002)	-0.008^c^ (0.002)	0.004 (0.005)	-0.008^b^ (0.003)	-0.013^c^ (0.004)	-0.009^b^ (0.003)	-0.009^c^ (0.002)	-0.004^c^ (0.001)
Group 3: third quartile	-0.014^c^ (0.003)	-0.009^c^ (0.002)	-0.006^c^ (0.002)	-0.009^c^ (0.002)	-0.002 (0.005)	-0.011^c^ (0.003)	-0.005 (0.004)	-0.010^c^ (0.003)	-0.006^c^ (0.002)	-0.004^c^ (0.001)
Group 4: top quartile	-0.010^c^ (0.003)	-0.009^c^ (0.003)	-0.002 (0.003)	-0.006^c^ (0.002)	0.001 (0.006)	-0.007^a^ (0.004)	-0.008^a^ (0.005)	-0.004 (0.004)	-0.005^a^ (0.003)	-0.006^c^ (0.001)
Patient Groups Based on Quartiles of Zip Code Level Median Household Income										
Group 1: lowest quartile	-0.008^c^ (0.003)	-0.005^b^ (0.002)	-0.002 (0.002)	-0.004^c^ (0.002)	0.005 (0.005)	-0.008^b^ (0.003)	-0.005 (0.004)	-0.006^a^ (0.003)	-0.005^b^ (0.002)	-0.004^c^ (0.001)
Group 2: second quartile	-0.010^c^ (0.003)	-0.007^c^ (0.002)	-0.004^a^ (0.002)	-0.007^c^ (0.002)	0.004 (0.005)	-0.006^a^ (0.003)	-0.011^b^ (0.005)	-0.008^b^ (0.004)	-0.007^c^ (0.002)	-0.003^c^ (0.001)
Group 3: third quartile	-0.015^c^ (0.003)	-0.011^c^ (0.002)	-0.008^c^ (0.002)	-0.011^c^ (0.002)	-0.005 (0.005)	-0.014^c^ (0.003)	-0.010^b^ (0.004)	-0.010^c^ (0.004)	-0.010^c^ (0.002)	-0.005^c^ (0.001)
Group 4: top quartile	-0.013^c^ (0.003)	-0.009^c^ (0.002)	-0.005^b^ (0.002)	-0.008^c^ (0.002)	-0.006 (0.005)	-0.009^c^ (0.003)	-0.007^a^ (0.004)	-0.006^a^ (0.004)	-0.006^c^ (0.002)	-0.004^c^ (0.001)
Patient Groups Based on Quartiles of Elixhauser Mortality Index Scores										
Group 1: lowest quartile	-0.012^c^ (0.002)	-0.004^b^ (0.002)	-0.000 (0.002)	-0.006^c^ (0.002)	0.002 (0.005)	-0.009^c^ (0.002)	-0.005 (0.004)	-0.004 (0.003)	-0.005^c^ (0.002)	-0.002^c^ (0.000)
Group 2: second quartile	-0.009^c^ (0.003)	-0.005^b^ (0.002)	-0.002 (0.002)	-0.005^c^ (0.002)	-0.003 (0.006)	-0.008^b^ (0.003)	-0.010^b^ (0.004)	-0.003 (0.003)	-0.006^c^ (0.002)	-0.002^c^ (0.001)
Group 3: third quartile	-0.012^c^ (0.003)	-0.008^c^ (0.002)	-0.004^b^ (0.002)	-0.008^c^ (0.002)	-0.005 (0.004)	-0.005 (0.004)	-0.007^a^ (0.004)	-0.008^b^ (0.003)	-0.007^c^ (0.002)	-0.004^c^ (0.001)
Group 4: top quartile	-0.013^c^ (0.003)	-0.014^c^ (0.002)	-0.007^c^ (0.002)	-0.010^c^ (0.002)	0.003 (0.004)	-0.015^b^ (0.007)	-0.010^a^ (0.005)	-0.011^c^ (0.004)	-0.012^c^ (0.003)	-0.008^c^ (0.001)

Notes: Robust standard errors in parentheses. All standard errors are clustered at the hospital level

All models control for patient and hospital attributes and year fixed effects

^a^Significant at 0.10 level

^b^significant at 0.05 level

^c^significant at 0.01 level

For the sake of brevity, we present only the DD estimates, i.e., the coefficients of the interaction terms. The DD estimates for overall readmission rates (first row of Table 4) confirm our observations about general trends as well as findings in previous studies [7, 10, 14]. In the full sample of patients, a statistically significant decline in readmissions occurred for Medicare AMI patients (a reduction of 1.2% relative to Medicare GI patients and 0.9% relative to private insurance patients), Medicare HF patients (0.8% relative to Medicare GI and private insurance), Medicare PN patients (0.5% relative to Medicare GI and 0.8% relative to private insurance), and HRRP-targeted conditions overall (0.8% relative to Medicare GI and 0.7% relative to private insurance).

The results in Table 4 are somewhat encouraging when it comes to vulnerable populations. Relative to Medicare GI patients, the readmission rate reduction for Medicare AMI patients in low-income hospitals (Group 4 in the table) was a statistically significant 1.8% (highlighted in yellow in the table), larger than the 1.4% realized by high-income hospitals (Group 1 in the table). However, the readmission rate reductions for Medicare HF, PN, or All-Target patients relative to Medicare GI patients were larger for high-income hospitals than for low-income hospitals. Among the four hospital groups based on quartiles of Medicaid patients, the readmission rate reduction was generally larger for hospitals serving the largest (Group 4 in the table) or the second largest percentage (Group 3 in the table) of Medicaid patients.

The readmission rate reduction for Medicare AMI, HF, PN, or All-Target patients relative to Medicare GI patients is not equitable for low- and high-income patients, as high-income patients gained more than low-income patients. The largest reduction was in Group 3. This suggests that upper-middle income patients may have benefited more from the program than other income groups.

When it comes to patients’ comorbidities, high-risk patients in all index hospitalizations gained the most reduction in readmissions relative to Medicare GI patients.

We find similar patterns when comparing Medicare patients to patients with the same condition but with private insurance. The readmission rate for Medicare AMI patients admitted to low-income hospitals (Group 4 in the table) experienced the largest decline among the four income-based hospital groups. The reduction was the largest for upper-middle income patients and high-risk patients.

The gray columns in Table 4 report DD estimates for conditions that were not targeted by the HRRP. The DD estimates in the first gray column (with Medicare GI patients as the control group) indicate no significant differences in the changes in readmission rates between non-targeted conditions and GI conditions. However, the second gray column in the table shows significant differences in non-target condition readmissions between Medicare patients and private insurance patients 45+ years of age. The reductions were larger among vulnerable populations, including low-income and high-Medicaid hospitals as well as high-risk patients. Although the DD estimates were smaller in magnitude than those for the targeted conditions, these findings could be interpreted as positive spillover effects on readmissions that are not targeted by the HRRP among Medicare patients relative to the privately insured patients. This finding is consistent with Fig. 3, which showed that after 2012, the readmission rates for non-targeted conditions were decreasing at a faster rate among Medicare patients than privately insured patients.

### Sensitivity analysis

We carried out several robustness checks. First, we replaced the Elixhauser mortality index score with the 29 Elixhauser comorbidity indicators. The conclusions are comparable.

Second, in Additional files 1 and 2: Appendices A and B we estimated DDD models. The DD estimates from Table 4 could be biased if other factors unrelated to the HRRP affect the treatment group but not the control group. Suppose that there is another ‘shock’ only to Medicare patients with AMI, which is unrelated to the HRRP, and could reduce their readmissions. For example, such a ‘shock’ could be technological advances in the treatment of AMI that did not happen to GI conditions. Then the DD estimates would overstate the effect of HRRP. As a robustness check, we used the DDD method, which divides observations further into two subsets, a group that is more sensitive and a group that is less sensitive to the treatment.

In Additional file 1: Appendix A, we use the ‘at-risk’ thresholds to further divide the treatment and control groups [14]. That is, we compare each hospital’s 30-day readmission rates of the three index conditions in each year to the national average rate from the CMS Hospital Compare data from July 2005 to June 2008 (19.9 for AMI, 24.5 for HF and 18.2 for PN). If readmission rates for any of the targeted conditions are worse than national rates in a year, then a hospital is ‘at risk’ and is assumed to be more ‘sensitive’ to the HRRP policy. About 30% of the ‘low-income’ hospitals in our sample are at-risk hospitals.

Table A1 illustrates the DDD approach by comparing changes in readmissions for Medicare AMI and Medicare GI patients. Table A2 summarizes the DDD estimates from the linear probability models. Unlike the state-level data used in previous studies [7, 14], the hospital identifiers in NRD do not track the same hospital across the years, as mentioned earlier. Hence, a hospital’s ‘at-risk’ status is derived for each year. When a hospital reduces its readmission from above to below the national average, this hospital will switch from at-risk to not-at-risk in the following year. This means that the readmission reduction this hospital made last year will not be counted towards the at-risk group. Hence the DDD estimate may underestimate the effect of the HRRP, in which case the results would be a lower bound on the effect of the HRRP.

In Additional file 2: Appendix B, we tested an alternative DDD design given the identification issue of the ‘at-risk’ status in Additional file 1: Appendix A. For each index condition, we separated the sample into two groups based on insurance types: Medicare (age 65 and above) or private insurance (age 45 and above). Within each insurance group, we derived the DD estimate by comparing an index condition with the GI condition. We then took the difference between the two DD estimates to derive the DDD estimates. Table B1 illustrates this alternative DDD approach by comparing changes in readmissions for AMI and GI patients across Medicare and Private Insurance. Table B2 summarizes the DDD estimates from the linear probability models. We note that, although we control for patient attributes in the linear probability models, there could be other unobserved differences between Medicare patients aged 65 and above and privately insured patients age 45 and above that may affect the DDD estimates. In addition, the DDD model requires the assumption that the other ‘shock’ affects AMI patients with Medicare and private insurance equally. This assumption might not hold true. For example, if hospitals are more likely to adopt new technology for privately insured AMI patients (or Medicare AMI patients), then the DDD estimate will be underestimated (or overestimated).

The robustness checks by DDD do not necessarily confirm the strong effects of the HRRP across hospital and patient groups. However, they confirm significant effects of the HRRP on reducing Medicare AMI readmissions in low-income hospitals and hospitals that treat a high percentage of Medicaid patients.

## Discussion

We examined national-level data on 34 million hospitalizations in 27 states and used DD models to estimate the effects of the HRRP on readmission rates for three targeted conditions (AMI, HF and PN) relative to readmission rates for conditions not targeted by the HRRP (GI) as well as readmission rates for patients with these conditions but with private insurance.

We found that, overall, readmission rates decreased, and in most cases, favorably to vulnerable hospitals and patients. Specifically, readmission rates decreased more for high-risk patients than for low-risk patients, and, in the case of Medicare AMI patients, decreased more for Medicare patients from low-income hospitals than for Medicare patients from high-income hospitals. We also found spillover effects from HRRP on reducing readmission rates among Medicare patients for non-targeted conditions relative to private insurance.

Our findings from national-level data support findings using Virginia and Florida data respectively when it comes to Medicare AMI patients [7, 14]. Specifically, we find that overall improvements in readmission rates for Medicare AMI patients relative to Medicare GI patients or patients with private insurance are statistically significant. Our findings are inconclusive with regard to HF and PN readmissions among some hospital and patient groups, which are in line with previous findings that there is no significant change in HR and PN readmissions in Virginia [7]. Our main conclusions on the impact of HRRP on Medicare AMI readmission rates are robust as indicated by the results from two DDD frameworks presented in Additional files 1 and 2: Appendices A and B, despite the data-imposed limitation that the separation of hospitals into at-risk and not-at-risk categories needs to be done year-by-year.

We list the following additional caveats in the interpretation of the results. First, we do not have a way of observing the specific mechanisms that drive the readmission processes at the hospital level. We rely on secondary administrative data that is limited in terms of describing the clinical details of the hospitalization and the follow-up. As pointed out in interviews [24], physicians and hospital administrators are making various investments and efforts to reduce readmissions in both clinical and non-clinical settings such as connecting patients with nearby clinicians, collaborating with pharmacists for better drug management, arranging transportation to health care services, and follow-up at patients’ homes. Future studies need to examine and evaluate these efforts.

Second, our study does not explicitly consider the Medicaid expansion and its impact on insurance types, coverage rates, hospital payer-mix, patient composition, and physician supply [25–27]. Many patients could gain coverage under the expansion, and hospitals could adapt in unobservable ways to meet the new demands for medical care. These changes could affect readmission rates. Because Medicaid expansion took effect in 2014, our paper and other studies on HRRP [7, 8, 14, 28, 29] generally chose the sample period up to 2014 to avoid this potential problem. However, it is possible that hospitals, patients and insurance companies had already made changes during or before 2014. Some of the trends we observed could be influenced by these changes. An ideal solution and robustness check would be to use the event-study approach using stratified samples based on expansion and non-expansion status [26, 27]. Unfortunately, the NRD does not track hospitals across years, and hospital state information is not available [30, 31]. We are thus unable to stratify samples based on expansion status. Future research, using data with State information, should examine the effects of HRRP after taking into account the Medicaid expansion.

Third, there is a possibility of cream-skimming, i.e., hospitals taking on fewer risky patients after the HRRP. If cream-skimming were to occur, our estimates of the impact of HRRP would be biased, as some of the reductions in readmission rates could be attributed to cream-skimming. We cannot test this hypothesis or use the baseline severity in a hospital in our analysis because of the inability to track hospitals across years. For the same reason, some hospital attributes and their quartile groups can vary over time as a response to HRRP and other policies such as Medicaid expansion.

Studies based on patient data in Florida and Virginia did not find evidence that hospitals, in response to the HRRP, carried out adverse practices such as delaying admissions, treating patients with greater intensity, altering discharge status, and changing patients admitted to hospital according to their age, race/ethnicity, health status, and socioeconomic status [7, 14]. If hospitals did not make these changes, it is reasonable to assume that hospitals did not practice cream-skimming in response to the HRRP. Even if risky patients were redirected from a high-readmission-rate hospital to a low-readmission-rate hospital, this could still benefit patients and hospitals at the state or national level because, presumably, patients were treated in a higher quality hospital. Studies have found that hospitals in some systems performed better than other systems and stand-alone hospitals because of their ability to allocate resources better [32, 33]. It would be a problem if risky patients were never admitted by any hospital or if the travel time and other related expenses for patients increased. An important future research direction is to examine the possibility of cream-skimming and, if it did occur, how it would affect the evaluation of the HRRP and the implications for patients, caretakers, and policymakers.

## Conclusion

The important findings of this research are that there is evidence that there has been a decline in readmissions across the board, and in some cases with more substantial changes for vulnerable populations. Although previous studies using pre-HRRP data raised concerns that the policy might disproportionally penalize vulnerable hospitals and patients [2–4], post-HRRP studies found that the HRRP penalty did not significantly affect the financial performance of vulnerable hospitals in particular (e.g., safety-net hospitals [8]). Together with our findings, this suggests that HRRP appears to be creating the right incentives for reducing readmissions not only overall but also for vulnerable populations. Our study has implications for both future research and public health policy.

For future research, better data sources may further test and improve our conclusions. For instance, the insignificance of the DDD estimates for some conditions and populations indicates that more research is needed to identify whether the HRRP is driving these changes. Future research should rely on datasets that link hospital IDs across the pre- and the post- period, and should include investigating other possible reasons for the reduction, as well as studying the actions of health care providers once the patient has left the hospital. With linked hospital IDs across years, future studies can test the cream-skimming hypothesis, study the changes of hospital baseline characteristics, and include fixed effects in models. Finally, like many studies on this topic, we focus on the program’s short-term effect. As more data becomes available, it is important to examine the HRRP’s long-run effects and implications.

There are several policy implications both broad and limited to the HRRP. In the broad sense, the HRRP addresses a major problem in US healthcare by incorporating Pay-for-Performance (P4P) principles, and the fact that it does not impact vulnerable populations negatively is encouraging for applying a P4P framework to US healthcare. Unlike other developed countries such as Canada, UK, and Germany, the United States does not have universal health care. Rather, its health care system is fragmented and composed of both private and public programs. Individuals and families either apply for eligible public insurance such as Medicare, Medicaid and the State Children’s Health Insurance Program (SCHIP) or purchase private insurance plans through their employers or in the insurance market. The U.S. system is often criticized for two problems: affordability and inefficiency. Because private insurance programs are often expensive, especially insurance programs without subsidies from the government or employers, millions of Americans are uninsured. Compared to other countries, the U.S. spends the most on health care but often ranks far behind in health outcomes such as immunization rates, obesity and life expectancy [34].

The 2010 ACA was a major health care reform in the U.S. to address the two problems, and the HRRP is one of many programs to improve the efficiency in the U.S. health care system. The P4P model offers financial incentives to providers (e.g., physicians, hospitals, networks) in the form of penalties or rewards. Under the 2010 ACA, the Centers for Medicare and Medicaid Services launched many P4P programs, of which the most influential hospital programs are the HRRP, Hospital Value-Based Purchasing Program (HVBPP), and Hospital-Acquired Condition Reduction Program (HACRP).

As shown in our study, there are several challenges to evaluate the effectiveness of programs such as the HRRP. The outcomes can be based on the ICD codes in claims data like our study, or other reported outcome measure (e.g., patient surveys in the CMS Hospital Compare Program). These performance measures should reflect the quality of health care providers, and account for special circumstances such as high-risk patients, low-income areas or simply bad luck. Otherwise, hospitals and physicians will have less incentive to improve and disadvantaged populations could be negatively affected. Another challenge is the magnitude and design of the financial incentives such as what percentage of reimbursement should be used as rewards or penalties; should these percentages remain fixed or change over time; and whether penalties or rewards work better.

Our study explores a new aspect of previous analysis in the literature that suggests that the HRRP does work as it was originally designed. Not only has the HRRP made a difference for targeted populations and conditions, but it has also reduced readmissions for other non-targeted patients (e.g., AMI patients with private insurance). Even though the HRRP is only limited to Medicare patients, the spillover effect to other patients indicates that a government health care policy originally limited to certain patient groups may have far-reaching implications for other vulnerable populations. Even more encouraging is that studies have shown that hospitals do not carry out adverse practices as a response to the HRRP, such as delaying admissions, treating patients with greater intensity, altering discharge status, and changing patients admitted to hospital according to their age, race/ethnicity, health status, and socioeconomic status [7, 14]. A close examination of the HRRP such as its performance measures, financial incentive structure, and the roll-out process could provide suggestions for adjustment of current P4P programs and the design of future programs. As with any program evaluation, a long-term study of HRRP and similar programs is needed, because such programs could be effective in the short-run, but unsustainable, ineffective or even harmful (often due to unintended consequences) in the long-run.

These short- and long-term evaluations and close examinations are especially important nowadays given the uncertainty surrounding the ACA. Unlike other parts of the ACA, the HRRP and other P4P programs aim to improve outcomes without an increase in spending. This feature is attractive to lawmakers on both sides of the aisle. If the HRRP and other P4P programs are proven to be effective, they are likely to continue and build up the momentum towards a P4P-based health care system [35, 36]. And a close examination of both successful and unsuccessful programs can provide important lessons to accelerate this process.

Another policy implication is to analyze why HRRP worked better for some conditions and population groups (e.g., AMI among patients with high severity) than for others (e.g., HF and PN). Although we cannot analyze this issue in this study, it is possible that different behavior changes could contribute to these outcomes. For example, hospitals could have adopted patient-centered care (PCC) among heart surgery patients, which is the provision of care that is responsive to the values, beliefs, and preferences of an individual [37, 38]. Or they could have improved patient education and post-discharge experience [39, 40]. A better understanding of the mechanisms at work would help hospitals and shed light on how to adjust the HRRP and other similar programs.

Finally, in the context of long-term implementation of the HRRP and similar programs, adjustments may be needed to avoid negative consequences for disadvantaged populations. It has been seven years since the introduction of the HRRP, and three new conditions have been added in the targeted conditions, COPD, CABG, and THA/TKA. It is important to continue the research on HRRP’s impact among different hospital types and patient groups (e.g., hospital ownership [17], safety-net status [8], and patient characteristics) over this longer period. For example, although the HRRP penalties did not immediately affect safety-net hospitals’ margins [8], their long-term effect is unknown. To avoid penalties, hospitals need to make investment in various areas [24]. The penalties imposed on hospitals serving a large percentage of low-income and at-risk patients could limit their resources to make such investments. This, in turn, can lead to future penalties and a vicious cycle. Although hospitals did not conduct adverse practices towards patients up to 2014 [7, 14], it does not mean they will behave the same way in the long run. It is also important to examine whether the reductions in readmission rates are sustainable or whether hospitals have reached their limit.

The twenty-first Century Cures Act, signed into law in December 2016, to come into effect in fiscal year 2019, aims to remedy the patient and hospital difference to some extent by dividing hospitals into five peer groups where hospitals are compared to other hospitals with a similar proportion of patients who are eligible for both Medicare and Medicaid, rather than to a national average [41]. As our study confirms, the positive effect of the HRRP is not evenly distributed across vulnerable populations, and there could be societal benefits if the policy is adjusted for the socioeconomic status of the patients and the neighborhood. Evaluations of other aspects of the program are also being carried out by the CMS and the research community.

## Supplementary information

**Additional file 1.** Appendix A: Triple Difference (DDD) Framework and Robustness Check.

**Additional file 2.** Appendix B: Alternative DDD Analysis and Robustness Check.

## Data Availability

The data that support the findings of this study are available from the Healthcare Cost and Utilization Project (HCUP), but restrictions apply to the availability of these data, which were used under license for the current study, and so are not publicly available. Data are, however, available from the authors upon reasonable request and with permission of HCUP. HCUP is a family of health care databases and related software tools and products developed through a Federal-State-Industry partnership and sponsored by the Agency for Healthcare Research and Quality (AHRQ).

## Abbreviations

ACA: Patient Protection and Affordable Care Act
AMI: Acute myocardial infarction
CABG: Coronary artery bypass graft
CMS: Centers for Medicare and Medicaid Services
COPD: Chronic obstructive pulmonary disease
DD: Difference-in-difference
DDD: Triple difference
GI: Gastrointestinal condition
HCUP: Healthcare Cost and Utilization Project
HR: Heart failure
HRRP: Hospital Readmissions Reduction Program
ICD-9: International Classification of Disease, ninth revision
NRD: Nationwide Readmission Database
PN: Pneumonia
THA/TKA: Elective primary total hip arthroplasty and/or total knee arthroplasty (THA/TKA)
