# Supplementary material for: Impact of the Medicare hospital readmissions reduction program on vulnerable populations
Source: BMC Health Serv Res. 2019 Nov 14;19:837. doi: 10.1186/s12913-019-4645-5 (PMC6857270; doi:10.1186/s12913-019-4645-5)
Supplement: Supplementary file 1 — Additional file 1. Appendix A: Triple Difference (DDD) Framework and Robustness Check. [file 12913_2019_4645_MOESM1_ESM.docx]

**Appendix A: DDD Framework and Robustness Check**

As a robustness check for our results, we estimated the following DDD model with standard errors clustered at the hospital level. The sample for this model includes Medicare patients aged 65 and above:

$\begin{matrix} Readmission_{iht}=\beta_{0}+ \beta_{1} TREAT_{i}+\beta_{2} POST_{i}+\beta_{3} ATRISK_{ht}+\beta_{4} TREAT_{i} \times POST_{i} + \\ \beta_{5} TREAT_{i}\times ATRISK_{ht}+\beta_{5}POST_{i}\times ATRISK_{ht}+\beta_{6} TREAT_{i}\times POST_{i}\times ATRISK_{ht}+\theta X+\varepsilon_{iht} \end{matrix}$*--- (2)*

where the definitions of the variables *Readmission_iht_, TREAT_i_,* and *POST_i_* are the same as in equation (1) in the main body of the paper, and the dummy variable *ATRISK_ht_* equals one if hospital *h* is at risk for any penalty in year *t*. The DDD estimate can be obtained as the coefficient *β_6_* in front of the variable *TREAT_i_×POST_i_×ATRISK_ht_*

Table A1 illustrates the use of this DDD approach to compare changes in readmissions for Medicare AMI and Medicare GI patients. The implicit assumption is that Medicare AMI patients in ‘at-risk’ hospitals, i.e., panel A in Table A1, are subjected to the consequences of the HRRP policy as well as the additional shock; whereas Medicare AMI patients in the ‘not-at-risk’ hospitals, i.e., panel B, are only subjected to the additional shock. Medicare GI patients in both panels are not affected by either the HRRP policy or the additional shock. Under this assumption, the DD estimate in panel A, -0.013, includes the effects of both HRRP and the other shock. The DD estimate in panel B, -0.011, includes only the effect of the other shock. The difference between the two DD estimates, i.e., the DDD estimate of -0.002, should extract only the effect of the HRRP.

Table A2 summarizes the DDD estimates from the linear probability models. The DDD estimates are mostly insignificant. The most significant reductions in readmission rates relative to a non-targeted condition (GI) are for Medicare AMI patients, especially those at low-income hospitals (a reduction of 2.8%) and high-Medicaid hospitals (a reduction of 1.3%). It also appears that Medicare AMI patients in the lowest income quartile fared better than Medicare AMI patients in the highest income quartile (a reduction of 0.9% in readmission rates versus a reduction of 0.4%). Medicare AMI patients of low socioeconomic status also appear to do well relative to private insurance AMI patients.

There does not appear to be a significant improvement in readmission rates for high-risk patients (the bottom row in Table A2). There is some evidence that readmission rates for Medicare AMI patients and Medicare All Target condition patients relative to private insurance patients with the same conditions have improved for patients with scores in the third quartile of the Elixhauser Index.

The DDD estimates in the two gray columns of Table A2 can be used to confirm the observations from the DD analysis. The DDD estimates in the first gray column are mostly negative and statistically insignificant. The DDD estimates in the second gray column are negative and statistically significant for vulnerable populations, indicating that there may be some spillover effects of the HRRP on patient readmissions for conditions that are not targeted by the HRRP relative to patients with private insurance.

We reran the DDD models for other definitions of the *ATRISK* variable, where the readmission rates were calculated not for all patients [14] but over only Medicare patients, as well as over Medicare patients with the specific condition. The DDD estimates are slightly different; however, they remain significant for Medicare AMI patients vs. Medicare GI and private insurance patients for hospitals serving a large percentage of low-income patients.

Table A1. DDD approach illustration with Medicare AMI patients as the treatment group, Medicare GI patients as the control group, and a further separation of patients from hospitals at risk and not at risk for HRRP penalties.

| Condition/Year | Before HRRP |  | After HRRP |  | Difference Over Time |
| --- | --- | --- | --- | --- | --- |
|  |  |  |  |  |  |
| A. Patients treated at hospitals at risk for penalties | |  | |  | |
| Medicare AMI | 0.204 |  | 0.186 |  | -0.018*** |
|  | (0.002) |  | (0.001) |  | (0.002) |
|  | N=66,634 |  | N=82,192 |  | N= 148,826 |
|  |  |  |  |  |  |
| Medicare GI | 0.143 |  | 0.137 |  | -0.006*** |
|  | (0.001) |  | (0.001) |  | (0.001) |
|  | N=231,416 |  | N=265,001 |  | N= 496,417 |
|  |  |  |  |  |  |
| Difference over conditions | 0.061*** |  | 0.049*** |  |  |
|  | (0.002) |  | (0.001) |  |  |
|  | N=298,050 |  | N=347,193 |  |  |
|  |  |  |  |  |  |
| Difference-in-Difference | -0.013*** | | |  | |
|  | (0.002) | | |  | |
|  | N=645,243 | | |  | |
|  |  | |  |  | |
| B. Patients treated at hospitals NOT at risk for penalties | | | | | |
| Medicare AMI | 0.157 |  | 0.145 |  | -0.012*** |
|  | (0.001) |  | (0.001) |  | (0.001) |
|  | N=117,608 |  | N=220,102 |  | N=337,710 |
|  |  |  |  |  |  |
| Medicare GI | 0.125 |  | 0.124 |  | -0.001* |
|  | (0.001) |  | (0.0005) |  | (0.001) |
|  | N= 289,253 |  | N=534,686 |  | N=823,939 |
|  |  |  |  |  |  |
| Difference over conditions | 0.031*** |  | 0.021*** |  |  |
|  | (0.001) |  | (0.001) |  |  |
|  | N=406,861 |  | N=754,788 |  |  |
|  |  |  |  |  |  |
| Difference-in-Difference | -0.011*** | | |  |  |
|  | (0.001) | | |  | |
|  | N=1,161,649 | | |  | |
|  |  | |  |  | |
| DDD | -0.002 | | |  | |
|  | (0.003) | | |  | |
|  | N=1,806,892 | | |  | |
| Notes: Unclustered standard errors are reported in parentheses.  * significant at 0.10 level; ** significant at 0.05 level; *** significant at 0.01 level | | | | | |

Table A2. DDD estimates for various combinations of treatment and control groups and vulnerable populations.

| **Sample** | **Treatment: Medicare 65+; Control: Medicare GI 65+** | | | | |  | **Treatment: Medicare 65+; Control: Private Insurance 45+** | | | | |
| --- | --- | --- | --- | --- | --- | --- | --- | --- | --- | --- | --- |
|  | **AMI** | **HF** | **PN** | **Target** | **NonTarget** |  | **AMI** | **HF** | **PN** | **Target** | **NonTarget** |
| **Full Sample** | -0.002 | -0.000 | 0.004* | 0.001 | -0.004 |  | -0.008** | -0.006 | -0.000 | -0.004* | -0.003** |
|  | (0.003) | (0.002) | (0.002) | (0.002) | (0.005) |  | (0.004) | (0.005) | (0.004) | (0.003) | (0.001) |
| **Hospital Groups Based on Quartiles of Low-Income Patients** | | | | | |  |  | | | | |
| Group 1: | 0.001 | -0.002 | 0.005 | 0.002 | -0.013 |  | 0.000 | -0.014 | 0.003 | -0.002 | -0.001 |
| lowest quartile | (0.007) | (0.004) | (0.005) | (0.003) | (0.010) |  | (0.009) | (0.010) | (0.007) | (0.005) | (0.002) |
|  |  |  |  |  |  |  |  |  |  |  |  |
| Group 2: | 0.000 | 0.000 | 0.010** | 0.004 | -0.010 |  | -0.006 | -0.001 | 0.007 | -0.002 | -0.002 |
| second quartile | (0.006) | (0.005) | (0.004) | (0.003) | (0.009) |  | (0.007) | (0.009) | (0.008) | (0.005) | (0.002) |
|  |  |  |  |  |  |  |  |  |  |  |  |
| Group 3: | 0.000 | -0.005 | -0.002 | -0.003 | -0.001 |  | -0.011* | -0.011 | -0.012 | -0.012** | -0.004* |
| third quartile | (0.005) | (0.004) | (0.004) | (0.003) | (0.008) |  | (0.006) | (0.008) | (0.007) | (0.005) | (0.002) |
|  |  |  |  |  |  |  |  |  |  |  |  |
| Group 4: | -0.028** | 0.007 | 0.002 | 0.001 | 0.014 |  | -0.022* | -0.002 | -0.004 | -0.001 | -0.003 |
| top quartile | (0.012) | (0.006) | (0.006) | (0.005) | (0.015) |  | (0.012) | (0.014) | (0.009) | (0.008) | (0.003) |
| **Hospital Groups Based on Quartiles of Medicaid Patients** | | | | | |  |  | | | | |
| Group 1: | -0.003 | -0.001 | 0.006 | 0.001 | 0.002 |  | -0.008 | -0.008 | -0.008 | -0.009 | -0.004** |
| lowest quartile | (0.008) | (0.005) | (0.005) | (0.004) | (0.011) |  | (0.009) | (0.011) | (0.009) | (0.006) | (0.002) |
|  |  |  |  |  |  |  |  |  |  |  |  |
| Group 2: | -0.000 | -0.002 | 0.003 | 0.001 | -0.013 |  | -0.011 | -0.014 | 0.010 | -0.005 | -0.000 |
| second quartile | (0.006) | (0.004) | (0.004) | (0.003) | (0.009) |  | (0.007) | (0.009) | (0.007) | (0.005) | (0.002) |
|  |  |  |  |  |  |  |  |  |  |  |  |
| Group 3: | 0.002 | 0.007 | 0.007* | 0.007** | -0.007 |  | -0.003 | 0.012 | 0.002 | 0.003 | -0.002 |
| third quartile | (0.006) | (0.004) | (0.004) | (0.003) | (0.010) |  | (0.007) | (0.009) | (0.007) | (0.005) | (0.002) |
|  |  |  |  |  |  |  |  |  |  |  |  |
| Group 4: | -0.013* | -0.006 | -0.004 | -0.008* | 0.003 |  | -0.014* | -0.017* | -0.011 | -0.011** | -0.005** |
| top quartile | (0.007) | (0.005) | (0.005) | (0.004) | (0.011) |  | (0.008) | (0.010) | (0.008) | (0.005) | (0.002) |
| **Patient Groups Based on Quartiles of Zip Code Level Median Household Income** | | | | | |  |  | | | | |
| Group 1: | -0.009 | 0.001 | 0.001 | -0.001 | 0.003 |  | -0.018** | -0.003 | -0.003 | -0.006 | -0.006*** |
| lowest quartile | (0.006) | (0.004) | (0.004) | (0.003) | (0.009) |  | (0.007) | (0.009) | (0.007) | (0.005) | (0.002) |
|  |  |  |  |  |  |  |  |  |  |  |  |
| Group 2: | 0.001 | -0.002 | 0.007 | 0.002 | 0.003 |  | -0.001 | -0.004 | -0.003 | -0.004 | -0.002 |
| second quartile | (0.006) | (0.004) | (0.004) | (0.003) | (0.010) |  | (0.008) | (0.010) | (0.008) | (0.005) | (0.002) |
|  |  |  |  |  |  |  |  |  |  |  |  |
| Group 3: | 0.005 | -0.002 | -0.003 | -0.001 | -0.003 |  | -0.005 | -0.014 | 0.001 | -0.007 | -0.002 |
| third quartile | (0.006) | (0.005) | (0.005) | (0.004) | (0.010) |  | (0.007) | (0.009) | (0.008) | (0.005) | (0.001) |
|  |  |  |  |  |  |  |  |  |  |  |  |
| Group 4: | -0.004 | 0.002 | 0.012** | 0.004 | -0.025** |  | -0.013 | -0.003 | 0.006 | -0.002 | -0.002 |
| top quartile | (0.007) | (0.005) | (0.005) | (0.004) | (0.010) |  | (0.008) | (0.010) | (0.008) | (0.005) | (0.002) |
| **Patient Groups Based on Quartiles of Elixhauser Mortality Index Scores** | | | | | |  |  | | | | |
| Group 1: | -0.004 | 0.003 | 0.007 | 0.002 | -0.005 |  | -0.007 | -0.005 | -0.000 | -0.003 | -0.002** |
| lowest quartile | (0.005) | (0.004) | (0.005) | (0.003) | (0.010) |  | (0.005) | (0.008) | (0.007) | (0.004) | (0.001) |
|  |  |  |  |  |  |  |  |  |  |  |  |
| Group 2: | 0.000 | -0.000 | 0.003 | 0.001 | -0.003 |  | 0.000 | -0.008 | -0.006 | -0.004 | -0.003** |
| second quartile | (0.006) | (0.005) | (0.005) | (0.004) | (0.012) |  | (0.007) | (0.009) | (0.007) | (0.005) | (0.001) |
|  |  |  |  |  |  |  |  |  |  |  |  |
| Group 3: | -0.005 | -0.000 | 0.006 | 0.001 | 0.002 |  | -0.031*** | -0.007 | 0.003 | -0.010** | -0.003** |
| third quartile | (0.006) | (0.004) | (0.004) | (0.003) | (0.009) |  | (0.010) | (0.009) | (0.007) | (0.005) | (0.002) |
|  |  |  |  |  |  |  |  |  |  |  |  |
| Group 4: | 0.008 | -0.002 | 0.002 | 0.001 | -0.008 |  | -0.006 | -0.004 | 0.002 | -0.000 | 0.000 |
| top quartile | (0.007) | (0.005) | (0.004) | (0.004) | (0.009) |  | (0.015) | (0.012) | (0.008) | (0.006) | (0.002) |
| Notes: Standard errors in parentheses. All standard errors are clustered at the hospital level. | | | | | | | | | | | |
| All models control for patient and hospital attributes and year fixed effects. | | | | | | | | | | | |
| * Significant at 0.10 level; ** significant at 0.05 level; *** significant at 0.01 level. | | | | | | | | | | | |
