# Supplementary material for: Impact of the Medicare hospital readmissions reduction program on vulnerable populations
Source: BMC Health Serv Res. 2019 Nov 14;19:837. doi: 10.1186/s12913-019-4645-5 (PMC6857270; doi:10.1186/s12913-019-4645-5)
Supplement: Supplementary file 2 — Additional file 2. Appendix B: Alternative DDD Analysis and Robustness Check. [file 12913_2019_4645_MOESM2_ESM.docx]

**Appendix B: Alternative DDD Analysis and Robustness Check**

Given the identification issue of the ‘at-risk’ status, we tested the following alternative DDD model. The sample for this model includes Medicare and privately insured patients:

$\begin{matrix} Readmission_{iht}=\beta_{0}+ \beta_{1} TREAT_{i}+\beta_{2} POST_{i}+\beta_{3} Medicare_{i}+\beta_{4}TREAT_{i}\times POST_{i} + \\ \beta_{5}TREAT_{i}\times Medicare_{i}+ \beta_{5}POST_{i}\times Medicare_{i}+ \beta_{6}TREAT_{i}\times POST_{i}\times Medicare_{i}+\theta X+\varepsilon_{iht} \end{matrix}$--- (3)

The variable definitions for *Readmission_iht_, TREAT_i_,* *POST_i_* and *X* are similar to the definitions in the main body of the paper. The value of the variable *Medicare_i_* is set to 1 if the patient’s insurance is Medicare; 0 if it is private insurance.

In effect, for each index condition, we separate the sample into two groups based on insurance types: Medicare or private insurance. Within each group, we derive the DD estimate by comparing an index condition with the GI condition. We then take the difference between the two DD estimates to derive the DDD estimates. Since the HRRP only focuses on Medicare patients, it should only affect the DD estimate, comparing an index condition with GI, in the Medicare group. The policy should not affect the DD estimate in the private insurance group. If, however, there is another shock affecting the index condition, DD estimates in both the Medicare group and the private insurance groups will capture it. By subtracting the DD estimate in the private insurance group from the DD estimate in the Medicare group, the DDD estimate will provide the effect of HRRP, net of other shocks to the index condition.

Consider the AMI condition. For Medicare patients (Panel A in Table B1), the AMI readmission rate decreased by 1.8 percentage points from 17.4% to 15.6%. During the same period, the GI readmission rate decreased by 0.5 percentage points from 13.3% to 12.8%. The difference between the two reductions (the DD estimate) is thus 1.3%. If the HRRP was the only factor causing this change, then the impact of the HRRP would be 1.3%. However, if there was another shock, such as technological improvements for AMI treatment in general, then the DD estimate would be overstated.

Consider now Panel B in Table B1. For private insurance, the HRRP should not affect the relative difference in the readmission rate changes between AMI and GI patients. Therefore, the DD estimate of -0.006 in Panel B will only capture the impact from the other shock. The difference between the two DD estimates, -0.007, will, therefore, remove the impact from other shocks and extract only the impact from the HRRP.

Table B2 shows the DDD estimates for the linear probability model presented above. The findings are similar to the main paper, but many more of the DDD estimates are statistically significant, indicating that the changes in readmission rates for several types of vulnerable populations may indeed be attributable to the HRRP.

These DDD estimates may suffer from some limitations. Although we control for patient attributes in the linear probability models, there could be other unobserved differences between Medicare patients aged 65 and above and privately insured patients age 45 and above. These differences will affect the DDD estimates. In addition, this DDD model requires the assumption that the other shock affects AMI patients with Medicare and private insurance equally. This assumption might not hold. For example, if hospitals are more likely to adopt new technology for privately insured AMI patients (or Medicare AMI patients), then the DDD estimate will be underestimated (or overestimated).

Table B1. DDD calculation example.

| Insurance Type/Year | Before HRRP | |  | | | After HRRP | |  | | Difference Over Time |
| --- | --- | --- | --- | --- | --- | --- | --- | --- | --- | --- |
|  |  | |  | | |  | |  | |  |
| A. Medicare patients | | |  | | | | |  | | |
| Medicare AMI | 0.174 | |  | | | 0.156 | |  | | -0.018*** |
|  | (0.001) | |  | | | (0.001) | |  | | (0.001) |
|  | N=184,242 | |  | | | N=302,294 | |  | | N= 486,536 |
|  |  | |  | | |  | |  | |  |
| Medicare GI | 0.133 | |  | | | 0.128 | |  | | -0.005*** |
|  | (0.0005) | |  | | | (0.0004) | |  | | (0.001) |
|  | N=520,669 | |  | | | N=799,687 | |  | | N=1,320,356 |
|  |  | |  | | |  | |  | |  |
| Difference over conditions | 0.041*** | |  | | | 0.027*** | |  | |  |
|  | (0.001) | |  | | | (0.001) | |  | |  |
|  | N=704,911 | |  | | | N=1,101,981 | |  | |  |
|  |  | |  | | |  | |  | |  |
| Difference-in-Difference | -0.013*** | | | | | | | |  | |
|  | (0.001) | | | | | | | |  | |
|  | N=1,806,892 | | | | | | | |  | |
|  |  | | |  | | | | |  | |
| B. Private insurance patients | | | | | | | | | | |
| Private AMI | 0.075 |  | | | 0.067 | |  | | -0.007*** | |
|  | (0.001) |  | | | (0.001) | |  | | (0.001) | |
|  | N=86,452 |  | | | N=135,714 | |  | | N=222,166 | |
|  |  |  | | |  | |  | |  | |
| Private GI | 0.088 |  | | | 0.087 | |  | | -0.001 | |
|  | (0.001) |  | | | (0.0005) | |  | | (0.001) | |
|  | N= 238,992 |  | | | N=339,656 | |  | | N=578,648 | |
|  |  |  | | |  | |  | |  | |
| Difference over conditions | -0.013*** |  | | | -0.019*** | |  | |  | |
|  | (0.001) |  | | | (0.001) | |  | |  | |
|  | N=325,444 |  | | | N=475,370 | |  | |  | |
|  |  |  | | |  | |  | |  | |
| Difference-in-Difference | -0.006*** | | | | | | | |  | |
|  | (0.001) | | | | | | | |  | |
|  | N=800,814 | | | | | | | |  | |
|  |  | | |  | | | | |  | |
| DDD | -0.007*** | | | | | | | |  | |
|  | (0.002) | | | | | | | |  | |
|  | N=2,607,706 | | | | | | | |  | |
| Notes: Unclustered standard errors are reported in parentheses.  * significant at 0.1 level; ** significant at 0.05 level; *** significant at 0.01 level. | | | | | | | | | | |

Table B2. DDD estimate for various combinations of treatment and control groups and vulnerable populations.

| **Sample** | **Treatment: Medicare 65+ patients with an index readmission and GI readmission**  **Control: Private insurance 45+ patients with an index readmission and GI readmission** | | | | |
| --- | --- | --- | --- | --- | --- |
|  | **AMI** | **HF** | **PN** | **Target** | **NonTarget** |
| **Full Sample** | -0.006*** | -0.005* | -0.004** | -0.003** | 0.004 |
|  | (0.002) | (0.002) | (0.002) | (0.002) | (0.005) |
|  |  |  |  |  |  |
| **Hospital Groups Based on Quartiles of Low-Income Patients** | | | | | |
| Group 1: | -0.008* | -0.003 | -0.008* | -0.006* | 0.000 |
| lowest quartile | (0.004) | (0.005) | (0.004) | (0.003) | (0.010) |
|  |  |  |  |  |  |
| Group 2: | -0.007** | -0.005 | -0.009** | -0.006** | -0.005 |
| second quartile | (0.003) | (0.004) | (0.004) | (0.003) | (0.008) |
|  |  |  |  |  |  |
| Group 3: | -0.002 | -0.007* | 0.000 | -0.001 | 0.016* |
| third quartile | (0.004) | (0.004) | (0.004) | (0.003) | (0.008) |
|  |  |  |  |  |  |
| Group 4: | -0.025*** | 0.003 | 0.004 | 0.002 | 0.027 |
| top quartile | (0.007) | (0.008) | (0.006) | (0.005) | (0.018) |
|  |  |  |  |  |  |
| **Hospital Groups Based on Quartiles of Medicaid Patients** | | | | | |
| Group 1: | -0.008** | -0.005 | -0.005 | -0.005 | 0.014 |
| lowest quartile | (0.004) | (0.005) | (0.004) | (0.003) | (0.009) |
|  |  |  |  |  |  |
| Group 2: | -0.004 | -0.009* | -0.005 | -0.005 | 0.007 |
| second quartile | (0.004) | (0.005) | (0.004) | (0.003) | (0.009) |
|  |  |  |  |  |  |
| Group 3: | -0.010*** | -0.004 | -0.008** | -0.005* | -0.014 |
| third quartile | (0.004) | (0.004) | (0.004) | (0.003) | (0.009) |
|  |  |  |  |  |  |
| Group 4: | 0.000 | 0.000 | 0.004 | 0.003 | 0.017 |
| top quartile | (0.005) | (0.005) | (0.005) | (0.004) | (0.011) |
|  |  |  |  |  |  |
| **Patient Groups Based on Quartiles of Zip Code Level Median Household Income** | | | | | |
| Group 1: | -0.004 | -0.001 | -0.002 | -0.001 | 0.025** |
| lowest quartile | (0.004) | (0.005) | (0.004) | (0.003) | (0.010) |
|  |  |  |  |  |  |
| Group 2: | -0.000 | -0.005 | -0.002 | -0.001 | 0.001 |
| second quartile | (0.004) | (0.005) | (0.004) | (0.003) | (0.010) |
|  |  |  |  |  |  |
| Group 3: | -0.012*** | -0.007 | -0.007* | -0.007*** | -0.007 |
| third quartile | (0.004) | (0.005) | (0.004) | (0.003) | (0.009) |
|  |  |  |  |  |  |
| Group 4: | -0.008** | -0.005 | -0.004 | -0.004 | -0.005 |
| top quartile | (0.004) | (0.005) | (0.004) | (0.003) | (0.009) |
|  |  |  |  |  |  |
| **Patient Groups Based on Quartiles of Elixhauser Mortality Index Scores** | | | | | |
| Group 1: | -0.007** | -0.002 | -0.002 | -0.002 | 0.006 |
| lowest quartile | (0.003) | (0.004) | (0.004) | (0.002) | (0.009) |
|  |  |  |  |  |  |
| Group 2: | -0.004 | -0.006 | 0.002 | -0.002 | 0.007 |
| second quartile | (0.004) | (0.005) | (0.004) | (0.003) | (0.010) |
|  |  |  |  |  |  |
| Group 3: | -0.005 | -0.008* | -0.010** | -0.007** | -0.019** |
| third quartile | (0.005) | (0.005) | (0.004) | (0.003) | (0.009) |
|  |  |  |  |  |  |
| Group 4: | -0.004 | 0.001 | -0.000 | -0.001 | 0.020** |
| top quartile | (0.007) | (0.006) | (0.004) | (0.004) | (0.009) |
| Notes: Robust standard errors are in parentheses. All standard errors are clustered at the hospital level.  All models control for patient and hospital attributes and year fixed effects.  * significant at 0.10 level; ** significant at 0.05 level; *** significant at 0.01 level. | | | | | |
